# A facile one-pot γ-radiation formation of gum arabic-stabilized cobalt ferrite nanoparticles as an efficient magnetically retrievable heterogeneous catalyst

**DOI:** 10.1039/d5ra00651a

**Published:** 2025-03-24

**Authors:** Mohamad Bekhit, Adel. S. Orabi, Fatma mohamad, Kholoud M. Abou El-Nour

**Affiliations:** a Radiation Chemistry Department, National Center for Radiation Research and Technology, Egyptian Atomic Energy Authority Cairo Egypt mohammed_bakhit2006@yahoo.com; b Department of Chemistry, Suez Canal University, Faculty of Science Ismailia 41522 Egypt

## Abstract

Currently, there is a demand for an effective solution to address toxic pollutants in aqueous environments. Consequently, creating a cost-efficient and effective catalytic system with the added benefits of easy recovery from the medium and the ability to be reused is essential. In this study, gamma (γ) radiolysis as a simple and environmentally friendly process under ambient settings was used to successfully manufacture a nanocatalyst of cobalt ferrite nanoparticles (CoFe_2_O_4_ NPs) modified gum arabic (GA) as a nontoxic, biocompatible, and inexpensive biopolymer. The prepared GA-CoFe_2_O_4_ NPs were evaluated by using X-ray diffraction (XRD), transmission electron microscopy (TEM), Fourier-transform infrared spectroscopy (FTIR), energy dispersive X-ray (EDX) mapping, and vibrating sample magnetometer analysis. XRD analysis illustrates the formation of CoFe_2_O_4_ NPs through the appearance of the characteristic peaks. TEM analysis shows the spherical shape of CoFe_2_O_4_ NPs with an average particle size diameter ranging from 20 to 30 nm. FTIR analysis of GA-CoFe_2_O_4_ NPs confirmed both the functionalization of GA with the CoFe_2_O_4_ NPs and the appearance of the specific signal of CoFe_2_O_4_ NPs. The atomic ratio obtained from EDX analysis matches the stoichiometric ratio of cobalt ferrite. The GA-CoFe_2_O_4_ NPs exhibit an excellent magnetic response of saturation magnetization of 47.619 emu g^−1^. The prepared CoFe_2_O_4_NPs were then evaluated as a catalyst for the catalytic reduction of *p*-NP, MO dye, and a mixture of these pollutants. The results showed that CoFe_2_O_4_ NPs have high catalytic efficiency in the reduction of mono or mixed compounds. Furthermore, recycling of the CoFe_2_O_4_ NPs catalyst was also confirmed and it could be magnetically recovered and reused for at least six times with a good catalytic efficiency.

## Abstract 2

Currently, there is a demand for an effective solution to address toxic pollutants in aqueous environments.

## Graphical abstract

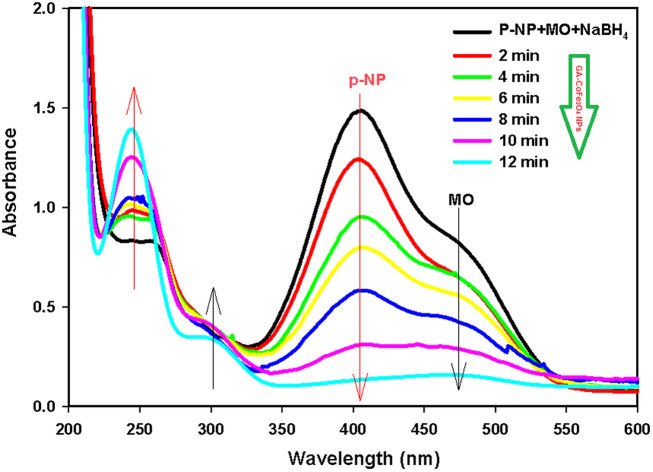

## 1. Introduction

Recently, the study of nanomaterials has expanded significantly due to their special chemical and physical characteristics, which make them suitable for a variety of uses in fields such as electronics, medicine, agriculture, and catalysis. Compared with bulk materials, nanoparticles have a lot of potential for usage as catalysts because of their high surface area-to-volume ratio.^1–4^

One of the major environmental problems is the contamination of the aquatic environment by the presence of toxic organic materials such as phenolic compounds and dyes. The contaminants of these compounds, even at trace levels, lead to adverse health effects in human beings and other living organisms. Nitrophenols, like *p*-nitrophenol (*p*-NP), pose a serious hazard to both humans and the environment because of their non-biodegradable, carcinogenic, and mutagenic qualities. They are employed extensively in a variety of industrial operations, such as the manufacturing of dyes, medications, herbicides, and pesticides. The transformation of *p*-NP compounds to amino phenols (*p*-aminophenol (*p*-AP)) by the catalytic reduction process holds great significance in the field of environmental remediation and is an important constituent in the manufacturing of different antipyretic and analgesic medications.^5^ On the other hand, the contamination of wastewater with dyes is detrimental to the ecosystem. Dyes are mutagenic and carcinogenic to aquatic life; they endanger human life at the bottom of the food chain. Numerous attempts have been made by researchers to eliminate harmful dyes from wastewater such as biological treatment,^6^ adsorption,^7^ membrane filtration,^8^ coagulation–flocculation,^9^ and catalytic processes.^10–12^ Among the different methods for wastewatertreatment, the catalytic reduction method has many benefits due to its simplicity, low cost and low energy consumption.^13^ Nowadays, the development of magnetic nanocatalysts with simple methods, high catalytic activity, and easy recovery processes has attracted enormous attention.

Researchers are very interested in magnetic nanoparticles (MNPs) because of their special characteristics, which include unique physicochemical properties and high magnetization values. Numerous sectors, including energy, environmental science, agriculture, biomedicine, biosensing, drug delivery, cancer research, bioimaging, bioseparation, and catalysis, have found extensive uses for magnetic nanoparticles. Because an external magnet can magnetize magnetic nanoparticles and isolate them from the reaction media, they are regarded as attractive catalysts. Because of its remarkable physical characteristics, cobalt ferrite (CoFe_2_O_4_) stands out among all other spinel ferrites (MFe_2_O_4_, where M stands for divalent metal ions like Zn, Ni, Mn, or Co). Cobalt ferrite is a type of spinel ferrite that possesses special magnetic properties, including high mechanical hardness, chemical stability, and coercivity.^14^

Magnetic nanomaterials are highly susceptible to aggregation and oxidation because of their large specific surface area, elevated chemical reactivity, and strong magnetic dipole interactions. Oxidation results in the formation of thin oxide layers that significantly change the properties of nanoparticles. Both aggregation and oxidation can reduce their catalytic effectiveness.^15^ The surface modification of magnetic nanoparticles by inorganic modifiers, surfactants, polymer molecules *etc.* improves the overall distribution of particles and their stability in aqueous media.^16,17^ Previous studies showed that silica coating is one of the popular methods in stabilizing CoFe_2_O_4_ NPs due to their excellent chemical resistance and ability to prevent aggregation. However, silica coating can slightly reduce the nanoparticles' magnetic response due to surface modifications that impact their magnetic interactions.^17,18^ Polymers are thought to be suitable host materials for the creation of nanoparticles due to their chemical stability and their functions as surface capping and stabilizing agents.^15–20^ The polymeric-modified CoFe_2_O_4_ nanoparticles exhibited excellent surface-active properties and the nanoparticles can be dispersed very well in the aqueous phase.^21^ The polyvinylpyrrolidone-coated CoFe_2_O_4_ nanoparticles nanoparticles showed well dispersion and homogeneous shape with narrow size distribution and negligible cytotoxicity.^22^ In the catalytic approaches, the surface coating and stabilization of magnetic nanoparticles improves their efficiency. Recently, Ali *et al.* (2020) synthesized CoFe_2_O_4_ nanospheres with and without surface modification and tested it in photo-driven catalytic remediation of hazardous dye (Alizarin Red S (ARS) dye). They concluded that the surface-modified CoFe_2_O_4_ nanospheres with triethylene glycol (PEG) exhibited high efficiency with better catalytic performance.^23^

It is crucial to keep in mind that the materials used in the pollution remediation procedure shouldn't be additional pollutants. In this regard, biopolymers are a remarkable and perfect option for this type of application.^24,25^ Biopolymers are biodegradable and derived from renewable resources and have no destructive effects on the environment. There has been a lot of interest in using environmentally friendly and non-toxic supports to synthesize heterogeneous catalysts. Metal catalysts supported by biopolymers demonstrated straightforward recyclability from the mixture reaction and could be reused multiple times without a significant decrease in their catalytic efficiencies.^26^

Polysaccharides are natural biopolymers with minimal toxicity, high biocompatibility, and biodegradability. Animals (glycogen, chitin, hyaluronan), plants (cellulose, starch, arabic gum), and microorganisms (curdlan, pullulan, dextran) are the sources of natural polysaccharides. Plants are among the most significant suppliers of polysaccharides when it comes to resources. Polysaccharides have several benefits such as safety, lack of toxicity, abundance in nature, biodegradability, hydrophilicity, good stability, cost-effective and many potential functionalization features because of the presence of free alcoholic and amine groups.^27,28^

Gum arabic is a biopolymer naturally obtained by exudation from *Acacia* trees. It is a complex polysaccharide mostly made up of galactose, arabinose, rhamnose, and glucuronic acid, with around 25% of its structure consisting of proteins. The location and age of the tree have an impact on the gum arabic's composition. The substance is used in a wide range of products, including beverages, confections, medications, baked goods, and cosmetics. Since it offers reactive functional groups and increases colloidal stability, it is a great dispersion for creating nanomaterials such as carbon nanotubes,^29^ nanogold,^30^ nanosilver,^31^ nanopalladium,^32^ nanoplatinum,^33^ bimetallic,^34^ metal oxides.^35–37^

The radiolytic strategy, which uses the reactive species (hydrogen radicals, and solvated electrons) created by radiolyzing the solvent itself (such as water, acetone, or alcohols) as reducing agents and causing particle nucleation, is one of the effective and successful ways to create nanoparticles. This method provides the special benefits that enable the synthesis of a range of nanomaterials, including: (1) simplicity because the reaction happens in one step at room temperature and pressure; (2) controllable shape and size of nanoparticles through the manipulation of the irradiation parameter such as dose or dose rate; (3) the active reducing species are created uniformly in the solution by radiolysis, eliminating the use of toxic reagents and consequently, the nanoparticles are prepared purely. (4) The scalability for large-scale synthesis can be easily executed.^38–42^ These major advantages make γ-radiosynthesis attractive from an environmental standpoint, where it has another sterilization function besides nanoparticle preparation.

Cobalt ferrite nanoparticles have been prepared by various methodologies such as the coprecipitation,^23,43^ sol–gel,^44,45^ hydrothermal,^46,47^ combustion method,^48^ polyol method,^49,50^ and radiation method.^51^ These methods take several steps of preparation with high-temperature treatment. The radiation method offers the one-pot, fast, controllable, and safe preparation of cobalt ferrite nanoparticles at room temperature without the need for additional reducing agents.

In the current study, a one-pot gamma radiation method was employed to synthesize CoFe_2_O_4_ NPs using gum arabic as a stabilizer. Using the X-ray diffraction technique (XRD), transmission electron microscopy (TEM), Fourier transform infrared spectrometer (FTIR), and vibrating sample magnetometer (VSM), the size, structure, and magnetic characteristics of the synthesized GA-CoFe_2_O_4_NPs have been determined. The catalytic reduction using GA-CoFe_2_O_4_NPs for *p*-NP, MO, and binary systems was studied.

## 2. Materials and methods

Gum arabic was purchased from Piochem Chemicals Co. (Egypt). Ferric chloride (FeCl_3_, M. Wt = 162.21 g mol^−1^) was obtained from Riedel-deHaën (Germany). Cobalt chloride hexahydrate (CoCl_2_·6H_2_O, M. Wt = 237.93 g mol^−1^) was obtained from Advent Chembio Pvt. Ltd (India). Isopropyl alcohol was obtained from Cario Erba Reaganti SPA (France). *p*-Nitrophenol was obtained from Loba Chemie (India). Sodium hydroxide pellets were purchased from Prolabo (Paris, France). Methyl orange was supplied from El Nasr Pharmaceutical Chemicals Company (Egypt).

### 2.1. Synthesis of GA-CoFe_2_O_4_ NPs

To prepare GA-CoFe_2_O_4_ NPs, a radiolysis method using gamma rays was employed. In a typical experiment, 1 g of gum arabic as a particle stabilizer was solubilized in 50 ml of bidistilled water using a magnetic stirrer at 70 °C to form a clear polymeric solution. After that, using the stoichiometry Fe/Co = 2 in the precursor solution, ferric chloride hexahydrate (FeCl_3_, 0.2 M) and cobalt chloride hexahydrate (CoCl_2_, 6H_2_O, 0.1 M) were dissolved separately, each one in 25 ml, and then added to the gum arabic solution. This solution was stirred for 1 h at room temperature, and the pH was adjusted to 12 using 5 M NaOH, producing a colloidal suspension of trivalent iron and cobalt hydroxides. After that, 10 ml of isopropanol as a hydroxyl radical scavenger was added to this dispersed solution with stirring for 30 min. Finally, the colloidal solution was exposed to 25 kGy gamma radiation irradiation (at room temperature and 0.8 kGy per h dose rate) using ^60^Co radiation facility present in National Centre for Radiation Research and Technology (NCRRT), Egyptian Atomic Energy Authority (EAEA), Egypt. CoFe_2_O_4_ nanoparticles were separated by filtration and washed several times with water and ethanol and left to dry at room temperature for 2 days. The final solid nanocatalyst was referred to as GA-CoFe_2_O_4_ NPs.

### 2.2. Nanoparticles characterization

GA-CoFe_2_O_4_ NPs were structurally, morphologically, and magnetically characterized using the following techniques: X-ray diffraction (XRD) analysis was performed by Shimadzu 6000 X-ray diffractometer using Cu Kα (1.5418 Å) radiation. The infrared spectroscopic analysis was carried out using an Attenuated Total Reflectance-Fourier transform infrared (ATR-FTIR) spectrometer (Bruker Vertex70, Germany). High-resolution Transmission electron microscopy (HRTEM) images were obtained with a JEOL JEM-2100 F microscope (Japan) worked at 200 kV. For the preparation of the TEM samples, drops of GA-CoFe_2_O_4_ NPs suspension in ethanol were supported onto a carbon-coated Cu grid. Elemental mapping and energy dispersive X-ray spectroscopy (EDX) analysis were obtained with a Scanning Electron Microscope (SEM), ZEISS EVO-15V coupled with an energy dispersive X-ray spectroscopy (EDX) probe. The magnetic properties were measured using a vibrating sample magnetometer instrument (VSM) (Lake Shore, Model-7410 Series VSM) at room temperature.

### 2.3. Catalytic reduction of *p*-NP, MO, and binary system

In a conventional quartz cell with a 1 cm path length, the catalytic reduction reaction of *p*-NP or (MO) by GA-CoFe_2_O_4_ NPs in the presence of excess NaBH_4_ was conducted. The catalytic process was evaluated as follows: 0.0189 g of NaBH_4_ was solubilized in 10 ml of cold (5 × 10^−5^ M) *p*-NP or MO solution (this means that the molar ratio of pollutant to NaBH_4_ is 1 : 1000). Finally, different weights of GA-CoFe_2_O_4_ NPs were added to a cuvette filled with 3 milliliters of pollutant and the catalytic performance was tracked spectrophotometrically by tracking the peak intensity change of pollutant at various time intervals using a Shimadzu UV 1800 spectrophotometer. On the other hand, to evaluate the effective capability of GA-CoFe_2_O_4_NPs against *p*-NP and MO Dye in a binary system, we taken 5 ml of (10 × 10^−5^ M) of *p*-NP and 5 ml of (10 × 10^−5^ M) MO solution and mixing it to form 10 ml of (5 × 10^−5^ M) of *p*-NP and MO solution and repeat the same catalytic steps.

## 3. Results and discussion

### 3.1. Characterization of GA-CoFe_2_O_4_ NPs

#### 3.1.1. XRD analysis

**Fig. 1:**
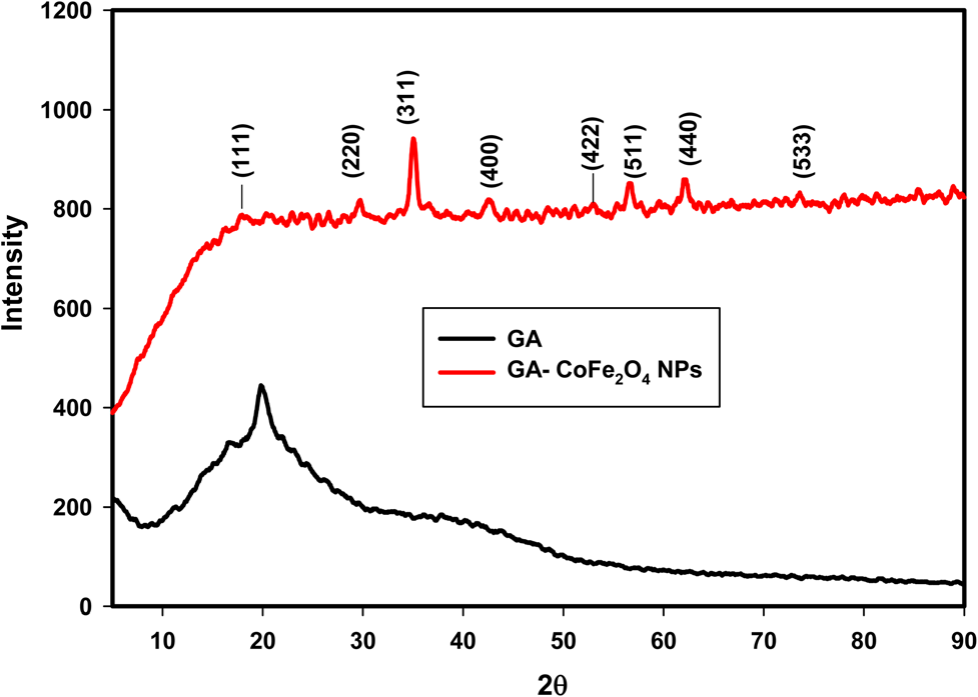
XRD patterns of GA and GA-CoFe_2_O_4_ NPs.

XRD analysis of nanomaterials is indispensable for determining their crystal structure and phase. Fig. 1 indicates the XRD patterns of GA and GA-CoFe_2_O_4_ NPs. The XRD spectrum of the gum arabic showed a distinctive strong and wide peak at 20° indicating the amorphous structure of gum arabic.^52^ On the other hand, the XRD pattern of GA-CoFe_2_O_4_ NPs demonstrated five new peaks at 29.7°, 34.9°, 42.5°, 56.5°, and 62.1°, which were attributed to the (220), (311), (400), (511), and (440) crystal planes, respectively. This result matches the spinel structure of the CoFe_2_O_4_ and is well in line with standard JCPDS card no. 022-1086,^53–55^ indicating that CoFe_2_O_4_ NPs were successfully prepared by gamma radiation-induced reduction. The average crystallite size (*D*) of CoFe_2_O_4_ was calculated by using the Scherrer equation (*D* = 0.9*λ*/*β* cos *θ*) where *λ* is the X-ray wavelength, *β* is the full width at half maxima of the most intense diffraction peak (311), and *θ* is the angle of this diffraction peak. The calculated crystallite size (*D*) of GA-CoFe_2_O_4_ NPs is 12.4 nm.

The mechanistic formation of GA-CoFe_2_O_4_ NPs by radiolytic method can be verified as follows: when the aqueous solution was exposed to ionizing radiation (gamma rays), both hydrated electrons (e_aq_^−^) and hydrogen radicals (H˙) as a reducing agents were formed as product of the radiolysis of water (eqn (1)).^56^ Under basic conditions, the H˙ radicals are scavenged by the OH^−^ anions and are replaced by e_aq_^−^ (eqn (2)). Hydrated electrons are an effective reducing agent that has a standard potential of *E*° = −2.87 V_NHE_ and can encourage the reduction process. It is important to note that hydroxyl radicals (OH˙), as oxidizing agents, also form during water radiolysis. So, isopropanol (CH_3_)_2_CHOH) is added to the reaction mixture as an OH˙ radical scavenger before irradiation. Fortunately, isopropanol scavenge OH˙ and form isopropyl radicals ((CH_3_)_2_C·OH) with reducing characters (eqn (3)).1
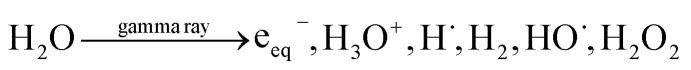

2 HO^−^ + H· → e_eq_^−^ + H_2_O

3 HO· + (CH_3_)_2_CHOH → (CH_3_)_2_C·OH + H_2_O

In a solution comprising Co(ii) and Fe(iii), the cobalt ions transition from Co(ii) to Co(iii) under aerated basic conditions (NaOH). When this mixed hydroxide solution is exposed to γ-radiation and the creation of hydrated electrons, the Co(iii) ions are then uniformly reduced back into Co(ii) inside the particles (eqn (4)) with the probability of ferric hydroxide reduction (eqn(5)).^51^

4 Co(OH)_3_, 2(Fe(OH)_3_) + e_eq_^−^ → Co(OH)_2_ + 2(Fe(OH)_3_) +HO^−^

5 Co(OH)_3_, 2(Fe(OH)_3_) + e_eq_^−^ → Co(OH)_3_ + Fe(OH)_2_+ Fe(OH)_3_ + HO^−^

Because cobalt ions with valency II are more stable than iron ions, an internal electron transfer from Fe II to Co III takes place, and cobalt ions are reduced as a result (eqn (6)). After that, and in the presence of gum arabic as a stabilizer, the cobalt-ferric hydroxide loses water molecules gradually, and a black precipitate of gum arabic-cobalt ferrite nanoparticles (GA-CoFe_2_O_4_ NPs) is formed (eqn (7)).

6 Co(OH)_3_ + Fe(OH)_2_+ Fe(OH)_3_ → Co(OH)_2_ + 2Fe(OH)_3_

7 Co(OH)_2_ + 2Fe(OH)_3_ → Co(OH)_2_ + 2FeO(OH)_2_ + H_2_ → CoFe_2_O_4_ + 4H_2_O

#### 3.1.2. FTIR analysis

**Fig. 2:**
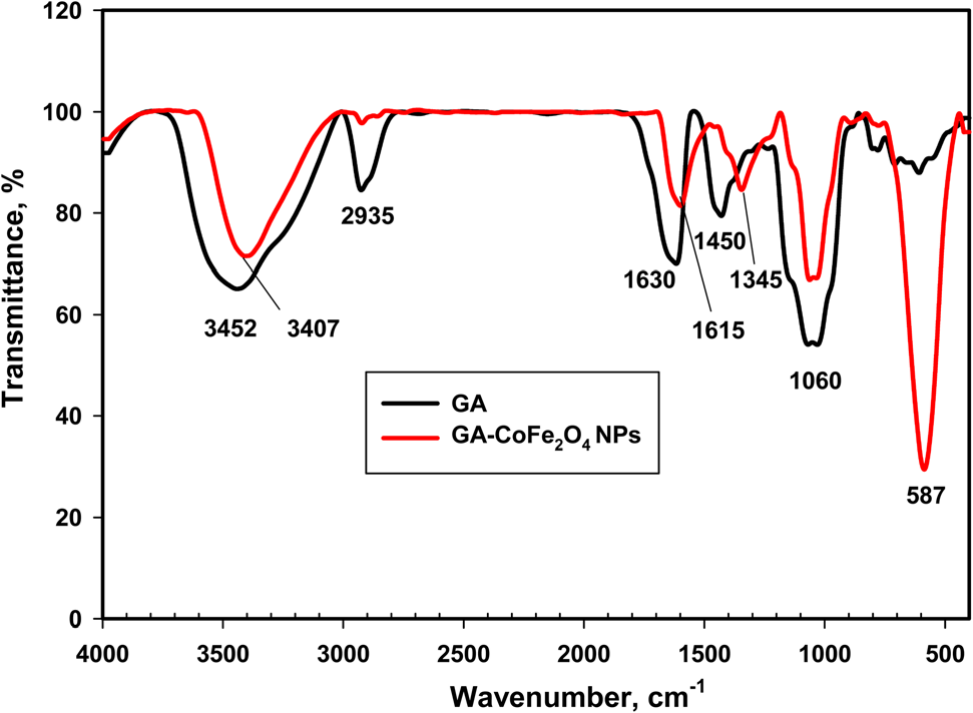
FTIR absorption spectra of GA-CoFe_2_O_4_ NPs.

FTIR study is performed to verify the attachment of GA on the CoFe_2_O_4_ NPs surface. The FTIR spectra of pure GA and GA-CoFe_2_O_4_ NPs are shown in Fig. 2. In the GA spectrum, a strongly broad band around 3452 cm^−1^ is ascribed to O–H stretching vibrations. The absorption peak in the 2935 cm^−1^ region is attributable to the stretching vibration of the C–H bonds. The peak at 1630 cm^−1^ is ascribed to the C=O stretching vibration and N–H bending vibration.^35,57^ The absorption band at 1450 cm^−1^ corresponds to the –OH bending of the acid group.^35^ The band at 1060 cm^−1^ corresponds to the characteristic C–O–C asymmetric stretching mode.^58^ On the other hand, the FTIR spectrum of GA-CoFe_2_O_4_ NPs exhibits the same feature of GA, but the intensity of the peaks was decreased and slightly shifted, confirming the functionalization GA with the CoFe_2_O_4_ NPs. This result indicates the crucial role of GA where it acts as a steric stabilizing agent and coats the CoFe_2_O_4_ NPs surface, avoiding the aggregation of CoFe_2_O_4_ NPs. Moreover, the absorption band observed at 587 cm^−1^ in the GA-CoFe_2_O_4_ NPs spectrum is characteristic of the magnetic CoFe_2_O_4_ and related to the metal–oxygen bonds.^46,54^

#### 3.1.3. TEM and EDX analysis

**Fig. 3:**
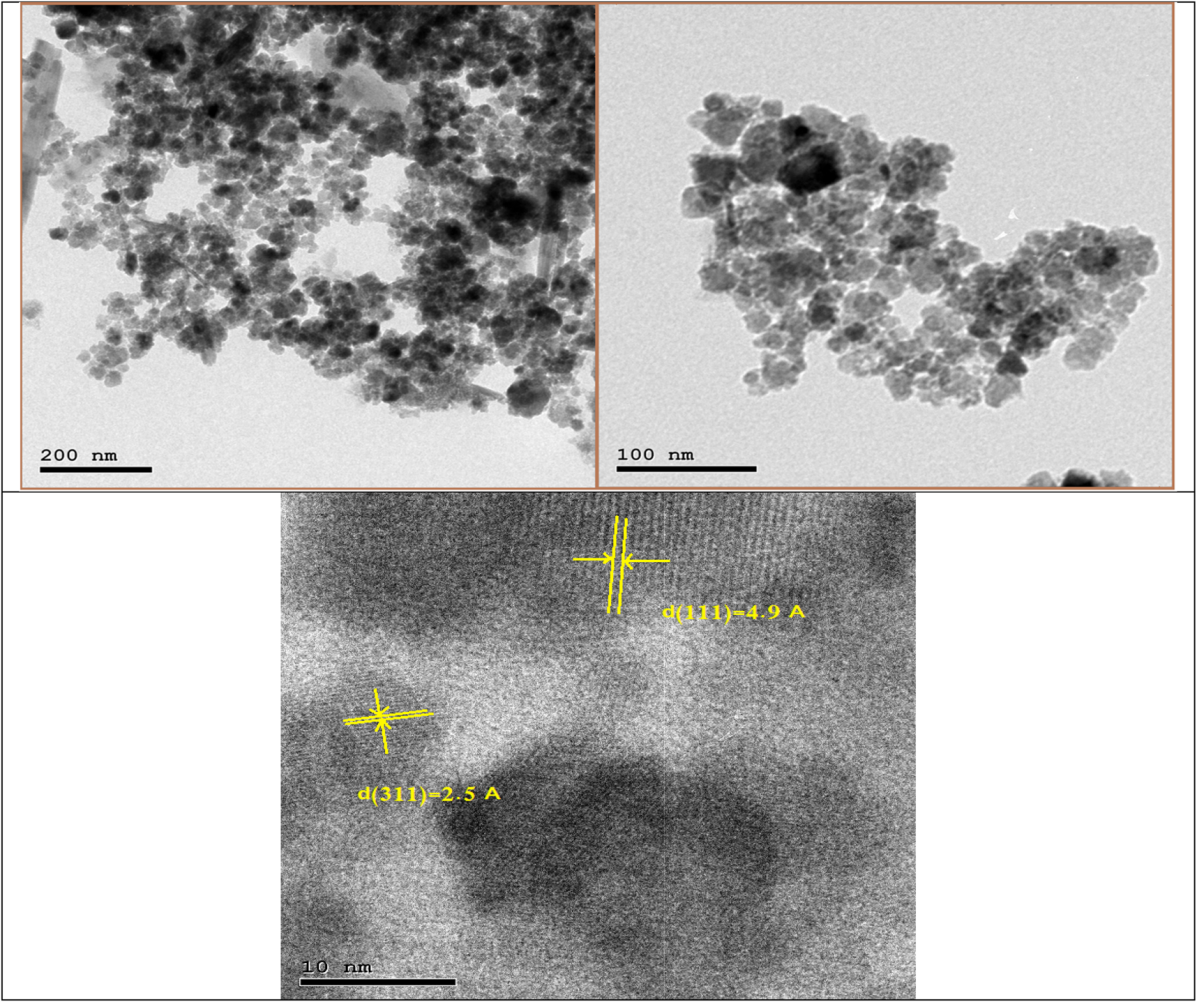
TEM image of GA-CoFe_2_O_4_ NPs with different magnifications.

To analyze the nanoparticles' size and shape, TEM images of the GA-CoFe_2_O_4_ NPs with different magnifications were presented in Fig. 3. We observed that there was a spherical and narrow particle size distribution of the synthesized GA-CoFe_2_O_4_ NPs, which signified the protective role of the stabilizing agent. The average size of GA-CoFe_2_O_4_ NPs ranged from 20 nm to 30 nm. It is obvious to note that the CoFe_2_O_4_ NPs appeared in a clustering form. This clustering is likely facilitated by strong dipole–dipole interactions between the magnetic nanoparticles, which may facilitate their clustering and aggregation.^43^ In the HRTEM image, the distances between the planes are *d*(111) = 4.9 A and *d*(311) = 2.5 A.

**Fig. 4:**
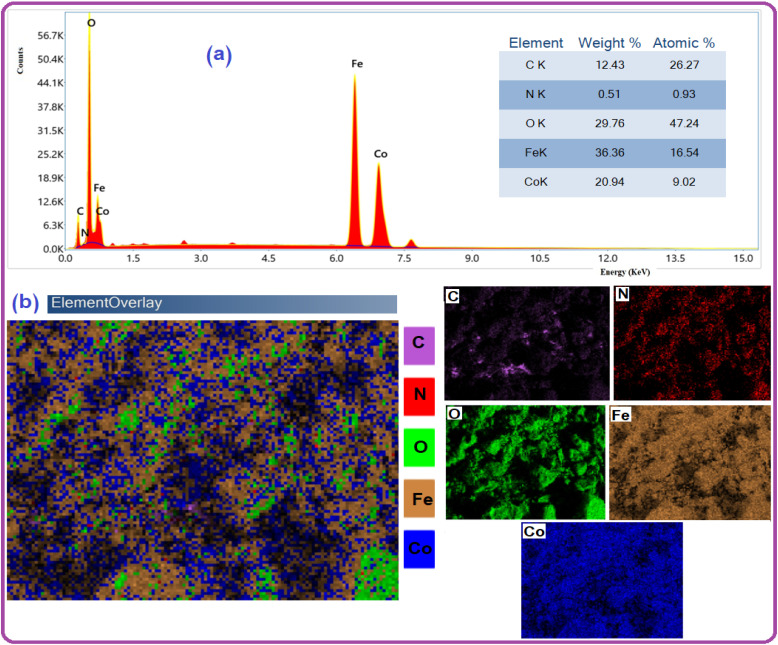
EDX spectra and their respective elemental mapping of GA-CoFe_2_O_4_ NPs.

Fig. 4a shows the energy-dispersive X-ray (EDX) spectra of the nanomaterials. The EDX spectrum affirmed the presence of C, N, O, Fe, and Co elements in the composite without the existence of any foreign elements, verifying the purity of the GA-CoFe_2_O_4_ NPs. As shown, peaks related to oxygen, iron, and cobalt have been found with atomic weight percentages equal to 47.24, 16.54, and 9.02%, respectively. The chemical compositional analysis of the samples by EDX shows the atomic ratio of Co to Fe is ∼0.5 which is apparent that the sample has the correct stoichiometric ratio of CoFe_2_O_4_.^59^ This proves the successful fabrication of the GA-CoFe_2_O_4_ NPs composite. Fig. 4b represented mapping analysis that recorded the existence of C, N, O, Fe and Co elements. As shown, these elements are homogeneously distributed on whole GA-CoFe_2_O_4_ NPs.

#### 3.1.4. VSM analysis

**Fig. 5:**
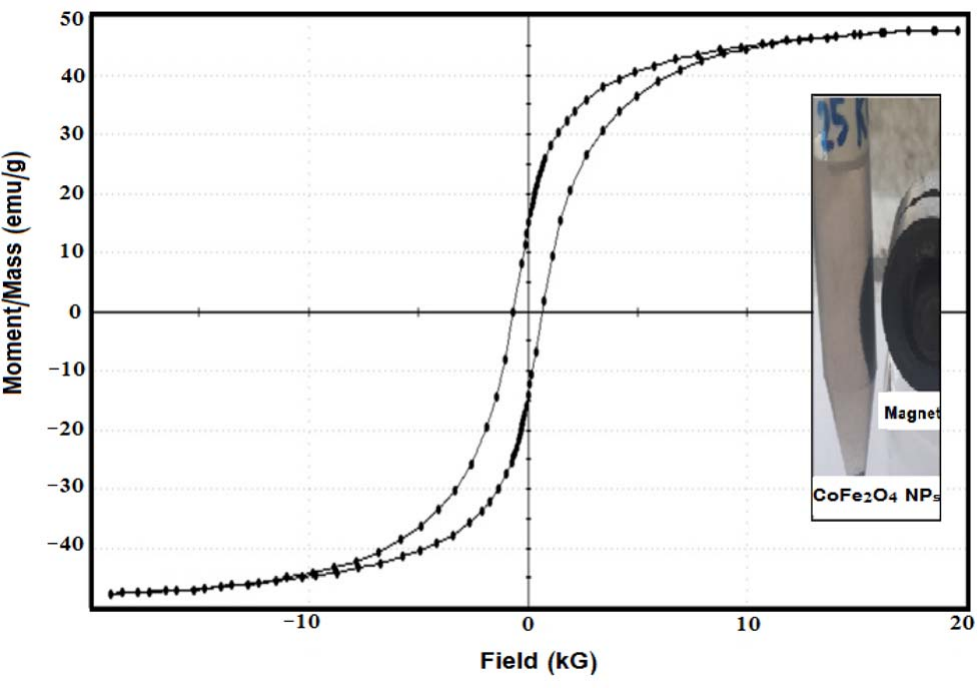
Magnetization curves of GA-CoFe_2_O_4_ NPs.

The magnetic hysteresis loop (M–H curve) of the GA-CoFe_2_O_4_ NPs obtained at room temperature is displayed in Fig. 5. It is evident from the M–H curve that GA-CoFe_2_O_4_ NPs possess a saturation magnetization (*M*_s_) equal to 47.619 emu g^−1^), indicating that they can be considered ferrimagnetic material and can be easily separated using an applied magnetic field as shown in the inset figure. Also, the GA-CoFe_2_O_4_ NPs had the following values: retentivity (Mr): 14.262 emu g^−1^ and coercivity (Hc): 656.34 G. The squareness ratio (SQ) is equal to Mr/Ms. When SQ is equal or greater than 0.5, the material has a single magnetic domain structure, whereas when it is below 0.5, it has a multi-domain structure. The calculated SQ value was 0.299. This value indicates the formation of a multi-domain structure, as observed previously.^59,60^

### 3.2. Catalytic activity of GA-CoFe_2_O_4_ NPs

**Fig. 6:**
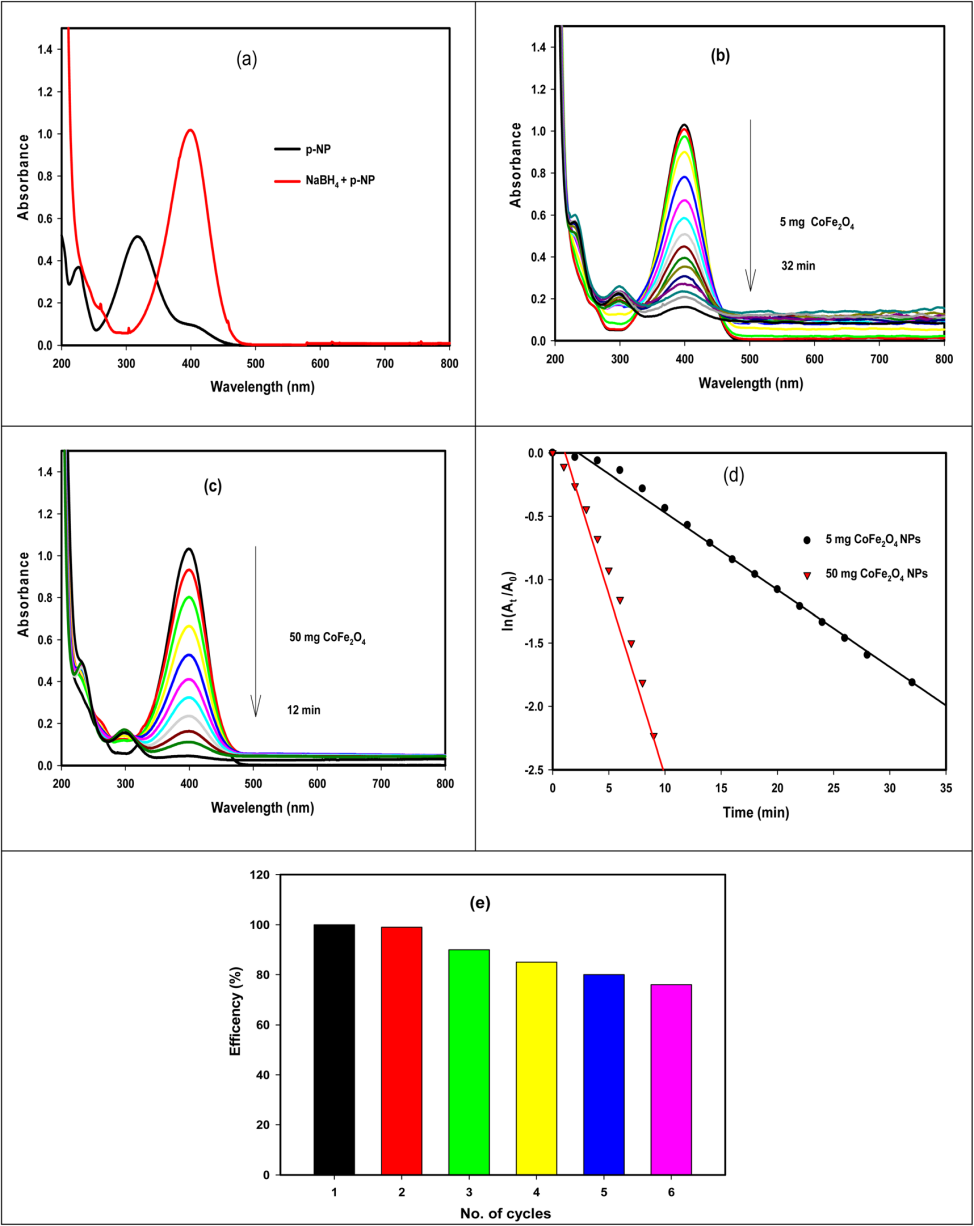
Reduction of *p*-NP into *p*-AP using GA-CoFe_2_O_4_ NPs catalyst: – (a) before and after adding NaBH_4_ solution. (b) and (c) Catalytic reduction using 5 mg and 50 mg of GA-CoFe_2_O_4_ NPs catalyst, respectively. (d) Plot of ln(*A*_*t*_/*A*_0_) *vs.* time. (e) GA-CoFe_2_O_4_ NPs catalyst reusability.

**Scheme 1:**
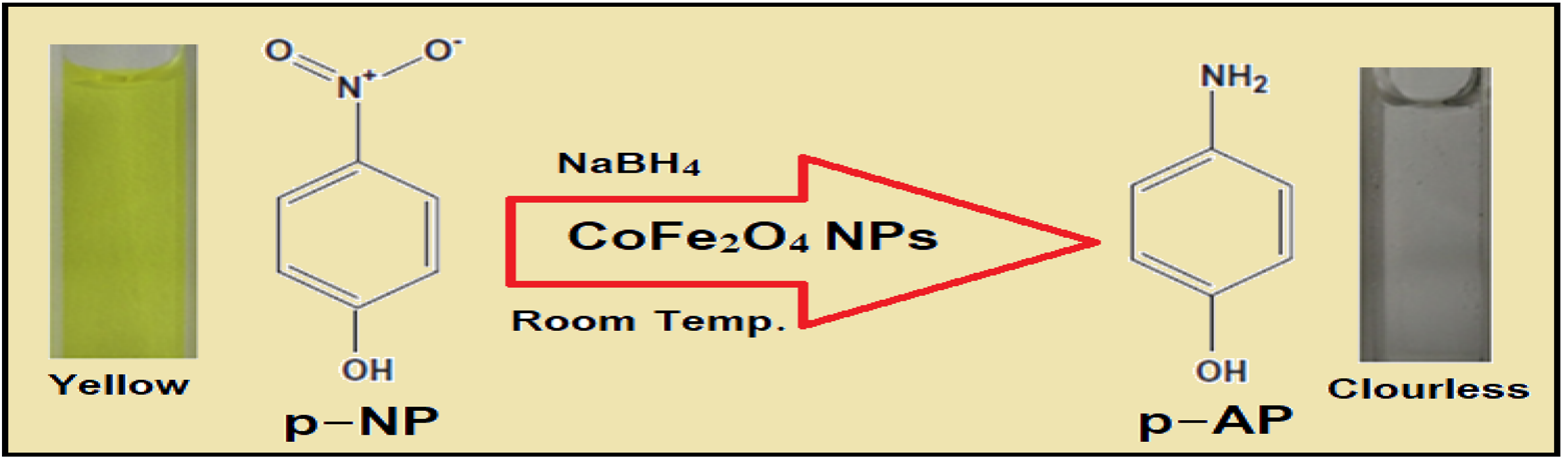
Reduction of *p*-NP with CoFe_2_O_4_ NPs catalyst.

The efficacy of the GA-CoFe_2_O_4_ NPs was tested in the catalytic reduction of *p*-nitrophenol (*p*-NP) into *p*-aminophenol (*p*-AP) and tracked using UV-vis spectroscopy. Fig. 6A displays the UV-vis spectra of *p*-NP solution with and without the addition of NaBH_4_. With the addition of NaBH_4_, the maximum absorbance (*λ*_max_) of *p*-NP (a bright yellow color) at 317 nm was shifted to 400 nm due to the construction of *p*-nitrophenolate ion (yellow color) in the solution.^61^ In the absence of GA-CoFe_2_O_4_ NPs, the yellow color of *p*-nitrophenolate did not change but upon the addition of GA-CoFe_2_O_4_ NPs as a catalyst, a gradual decrease in the yellow color of *p*-nitrophenolate occurred with time-associated with a gradual decrease in their absorption peak, indicating the remarkable progress in the reduction process of –NO_2_ group in *p*-NP into –NH_2_ group in *p*-AP (Scheme 1). Also, the emergence of a new and weak peak at 300 nm indicates the sign of the successful formation of *p*-aminophenol.^62^ It is observable that with that, the catalytic efficiency is increased, increasing the amount of GA-CoFe_2_O_4_ NPs where the degradation reaction was completed within 32 min after being catalyzed with 5 mg GA-CoFe_2_O_4_ NPs and 10 min with 50 mg GA-CoFe_2_O_4_ NPs at room temperature. The catalytic reduction reaction rate is independent of NaBH_4_ concentration and followed the pseudo-first-order kinetics.^63,64^ The rate constant values of *K* is the slope of the straight line of the linear relation between ln(*A*_t_/*A*_0_) and time *t*, where *A*_0_ and *A*_*t*_ are the absorption intensity at time 0 and *t*, respectively (Fig. 6d). The rate constant *K* is equal to 0.06 and 0.29 min^−1^ for the reaction catalyzed with 5 mg GA-CoFe_2_O_4_ NPs and 50 mg GA- CoFe_2_O_4_, respectively.

The increase in the catalytic efficiency with increasing nanocatalyst content is due to the increase in the surface area and, consequently, the high active site in the catalyst.^65^ The catalytic reduction route implies a chemisorption process of *p*-nitrophenolate ions on the surface of GA-CoFe_2_O_4_ NPs, and the reduction of *p-*nitrophenolate ions into *p*-aminophenol occurred by interfacial electron transfer released by BH^4−^ as an electron donor. *i.e.*, the catalytic reaction mechanism is facilitated by CoFe_2_O_4_ NPs, which act as an electron-transfer mediator that transports electrons to *p*-NP from sodium borohydride ion. Next, *p*-aminophenol desorbs from the CoFe_2_O_4_ NPs surface. The quicker the electron transfer process on the catalyst surface, the quicker the catalytic reaction.

To test if GA-CoFe_2_O_4_ NPs can be used several times, we carried out the reusability experiment by magnetic separation process and washing the catalyst with bidistilled water to be reused for 6th cycles, and the results are illustrated in Fig. 5d. The result revealed that the GA-CoFe_2_O_4_ NPs catalyst was reusable with a good result even after the six catalytic cycles. The decrease in response after each use is due to the catalyst's number of available active sites decreasing, as seen by the noticeable drop in response after each use.

**Fig. 7:**
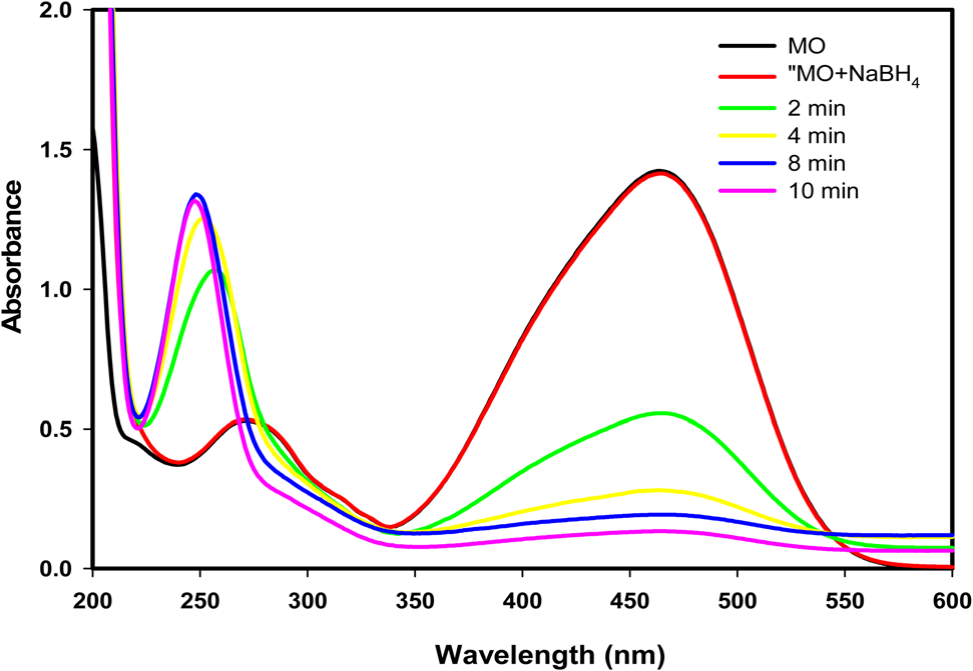
The UV-vis absorption spectra measured for the degradation of MO catalyzed by GA-CoFe_2_O_4_ NPs catalyst.

**Scheme 2:**
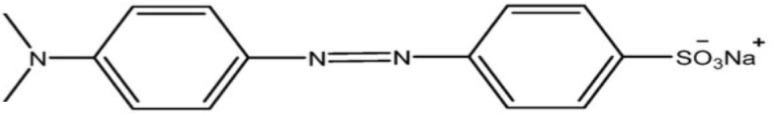
Chemical structures of MO dye.

Furthermore, the catalytic efficacy of GA-CoFe_2_O_4_ NPs was examined in the reduction of MO dye as a representation of well-known anionic azo dyes. As shown in Fig. 7, the MO dye has two absorption bands located at 270 and 464 nm qualified to the π → π* transition of aromatic rings and the conjugation mode of the azo band, respectively (Scheme 2). Upon using GA-CoFe_2_O_4_ NPs as catalyst, the characteristic MO dye absorption bands were decreased with the color diminish and new band appearance at located at 248 nm attributable to sulfanilic acid.^66^ The complete degradation of MO dye was taken 10 min at 50 mg of catalyst.

**Fig. 8:**
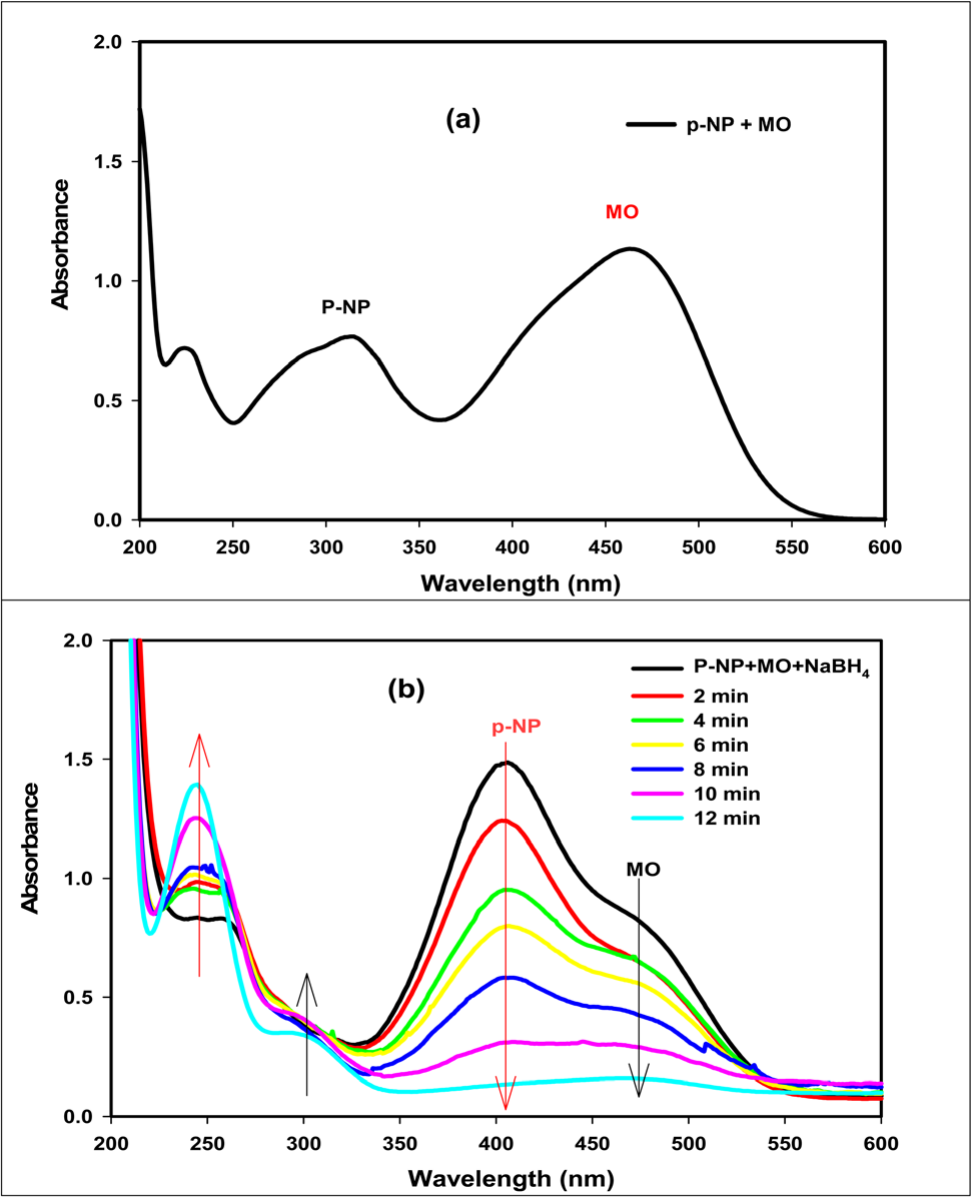
UV-vis absorption spectra of *p*-NP and MO mixture before (a) and after (b) using GA-CoFe_2_O_4_ NPs catalyst in presence of NaBH_4_ solution.

Since it is feasible to have contaminated waters with several contaminants, the reduction of organic pollutants in multi-pollutant systems is seen to be the most desired reaction.^67^ Thus, as a model reaction, we decided to investigate the reduction of *p*-NP and MO Dye in a binary system and the results are presented in Fig. 8. Fig. 8a shows the UV-vis spectra of the mixed *p*-NP and MO dye without the addition of NaBH_4_ having the two characteristic peaks of both *p*-NP and MO dye at 317 and 464 nm, respectively. After adding NaBH_4_ (Fig. 8b), the peak of *p*-NP shifted to 400 nm as mentioned above, and the peak belonging to MO dye appeared as a shoulder peak. After the addition of GA-CoFe_2_O_4_ NPs catalyst, a decrease in the intensity of these peaks with time occurred. The reduction of the *p*-NP and MO dye mixture was completed in 12 min. The results confirm that GA-CoFe_2_O_4_ NPs have remarkable catalytic degradation efficiency against both mono and multi-pollutant systems.

## 4. Conclusions

In this work, gamma radiolysis process was used to manufacture cobalt ferrite nanocatalyst modified with gum arabic as a safe biopolymer. Several analytical methods were used to evaluate the magnetic, morphological, and structural characteristics. TEM measurements showed that GA-CoFe_2_O_4_ NPs were uniformly shaped, spherically produced, and uniformly dispersed within the 20–30 nm range. With a saturation magnetization of 47.619 emu g^−1^, the VSM measurement of GA-CoFe_2_O_4_ NPs demonstrates a good magnetic response, making it suitable for magnetic separation. GA-CoFe_2_O_4_ NPs was applied to the reduction of *p*-NP and MO dye in both single and binary systems. The results revealed that the GA-CoFe_2_O_4_ NPs is an excellent catalyst for removing hazardous and toxic organic materials from waste solutions in both single and binary systems. Moreover, the GA-CoFe_2_O_4_ NPs showed good recyclable catalytic activity.

## Data availability

Data is contained within the article.

## Authors contribution

Mohamad Bekhit: data curation, investigation, methodology, writing – original draft writing – review & editing. Adel Orabi: data curation, investigation, writing, review & editing. Fatma Mohamad: data curation, investigation, methodology, writing, review & editing. Kholoud Abou El-Nour: data curation, investigation, methodology, writing – original draft writing – review & editing.

## Conflicts of interest

The authors declare no conflict of interest.
